# Nutrient Recovery from Municipal Wastewater for Sustainable Food Production Systems: An Alternative to Traditional Fertilizers

**DOI:** 10.1089/ees.2019.0053

**Published:** 2019-07-10

**Authors:** Ranjani B. Theregowda, Alejandra M. González-Mejía, Xin (Cissy) Ma, Jay Garland

**Affiliations:** ^1^ National Research Council (NRC) Post Doctoral Research, National Risk Management Research Laboratory, United States Environmental Protection Agency, Cincinnati, Ohio.; ^2^ Sêr Cymru National Research Network for Low Carbon, Energy and Environment, School of Environment, Natural Resources and Geography, Bangor, United Kingdom.; ^3^ National Risk Management Research Laboratory, United States Environmental Protection Agency, Cincinnati, Ohio.; ^4^ National Exposure Research Laboratory, United States Environmental Protection Agency, Cincinnati, Ohio.

**Keywords:** diammonium phosphate (DAP) fertilizer, emergy, nutrient recovery, struvite fertilizer, system analysis, system efficiency

## Abstract

Traditional wastewater management uses end-of-pipe approaches to remove pollutants in wastewater before discharge. Although effective in human health protection for decades, this approach of removal and disposal requires a high investment of energy and materials and overlooks the values of the key nutrients in wastewater such as phosphorus (P). Phosphorus in wastewater comes from the human metabolites of food, resulted from crop uptakes of fertilizer that ultimately derived from phosphate rock (PR). PR, however, could be depleted in this century, which would lead to a global food crisis. To address the question whether nutrient recovery is indeed a more efficient strategy from a system perspective and provides more benefits to society, this research compares fertilizer production from struvite to the traditional commercial fertilizers (e.g., diammonium phosphate, DAP). Emergy defined as the available energy required directly and indirectly through all transformations to make a product, process, or service is the tool used for system analysis in this study. Emergy accounting provides system analysis of total resource use and whole system efficiency. The results show that struvite production uses one order of magnitude less emergy than DAP production to produce one unit of fertilizer, indicating that struvite production is a more efficient process. This research sheds light on alternative nutrient management through nutrient recovery, which may achieve economic and environmental benefits and overall higher system efficiency.

## Introduction

### Importance of phosphorus and nitrogen for food production

Phosphorus (P) is essential to sustain all forms of life. Major process such as photosynthesis, energy transfer, cell division, growth, and quality of fruits and crops depend on this element. In animals, phosphorous is an important building block of DNA, RNA, and energy storage ATP, a major component of bones and teeth, and is associated with nutrition and growth as well. Therefore, it is vital for food production since it is one of three main components (with nitrogen [N] and potassium) in commercial fertilizers. Although phosphorous can be replenished by the fluxes of the global biogeochemical cycle through geological time, its major reservoir, phosphate rock (PR), is a nonrenewable resource due to its faster rate of use relative to its formation. Therefore, it directly affects world food security (Tyrell, 1999; Cordell *et al.*, 2011). According to a study focused on P reserves, if the current level of per capita PR production rate is applied to the median United Nation (UN) population projection, the current reserves would last until the year 2315. However, if population growth rate continues to stay similar to the current rate (as forecasted in the UN “high” population estimate), they would only last until 2170 (Daneshgar *et al.*, 2018). No substitute or synthetic version of concentrated P is available. Ultimately, P will have to be recycled at a large scale to avoid the collapse of agriculture. There is a great potential to recover it from human waste and transform it as a renewable resource and with a consistent supply (Guest *et al.*, 2009). Human urine itself contributes 80% of the total nitrogen and 40–50% of the total phosphate load to municipal wastewater (Wilsenach and Loosdrecht, 2006).

In contrast to P, nitrogen availability for fertilizer production is inexhaustible as the industrial process such as Haber–Bosch nitrogen fixation can convert nitrogen gas to ammonia; but ammonia synthesis is energy-intensive because so much energy is required to break the triple bond of molecular N ≡ N so that it can be converted (with the addition of hydrogen from water) to two molecules of ammonia. Ammonia production accounts for 90% of the nitrogen fertilizer production, consuming 1.2% of the world's total energy (International Fertilizer Industry Association, 2015). Production cost surveys show that natural gas costs consisted almost 70% of total ammonia production costs for North American producers (UNIDO/IFDC, 1998). Consequently, since year 2000, 26 of the 56 nitrogen fertilizer plants in the United States have shut down due to the higher cost of natural gas. Higher energy prices increase the cost of fertilizer, as well as an increase in food processing and distribution expenses (International Energy Agency, 2008).

Apart from the expense of the fertilizer, the environmental impacts of using fertilizers are also significant. If ammonium or P in the fertilizer is not used up by plants, it runs off and accumulates in groundwater and surface waters, leading to eutrophication. Eutrophication is a process of enrichment of water bodies with excess nutrients, and the subsequent decomposition of plant biomass exhausts the dissolved oxygen in water and significantly impairs water quality and biological structure and function. Existing eutrophication controls mostly centered on the reduction of the nutrient (P) loads to water bodies (Thornton, 1996; Dodds *et al.*, 2008). Also, although nitrogen is used to increase yield, it does not contribute to the buildup of soil fertility. Unbalanced use of nitrogen relative to other nutrients causes soil nutrient depletion. Nitrous oxide (N_2_O) from nitrification and denitrification processes is a greenhouse gas and may also contribute to the destruction of the stratospheric ozone when converted to nitric oxide (Byrnes, 1990). Agricultural emissions owing to N fertilizer use and manure management and emissions from natural soils represent 56–70% of all global N_2_O sources (Syakila and Kroeze, 2011). High energy use in fertilizer production exacerbates other environmental impacts such as climate change and air pollution.

In fertilizer application, the most common limiting factor is the availability of P. P is considered a low-cost commodity unevenly distributed around the globe with a market value that does not account for its ecological service or scarcity (Driver *et al.*, 1999). The orthophosphate form (H_2_PO_4_^−^) predominates as the form of P taken up by the plants. Ammonium phosphates (diammonium phosphate [DAP] and monoammonium phosphate [MAP]) are the leading forms of current phosphate fertilizer in the world (UNIDO/IFDC, 1998).

With increasing concerns about the long-term supply of rock phosphate and the eutrophication potential of phosphates in surface waters, it seems sensible to consider strategies that use wastewater as a new resource for consistent P acquisition and use (Vance *et al.*, 1999). It is well known that P and NH_4_-N naturally precipitate out from urine as struvite scale, causing clogging in wastewater piping networks (Ban and Dave, 2004). The alkalinity in the wastewater increases supersaturation of chemical species such as Mg^2+^, PO_4_^3−^, and NH_4_^+^, enhancing the natural precipitation of struvite causing scales on pipe walls and fittings that lead to increased friction and high turbulence. Recent efforts harvest struvite from different chemically based recovery processes (Guenther *et al.*, 2018). For example, struvite (MgNH_4_PO_4_•6H_2_O) derived from wastewater solid treatment processes (de-Bashan and Bashan, 2004) as well as from source-separated urine (Larsen *et al.*, 2009) can be operated at pilot and full commercial scales (Cullen *et al.*, 2013). It has been argued that the resource recovery can be achieved with the existing technologies, and new technological developments are on the horizon. It is important to balance the local/regional impacts and global goals (Guest *et al.*, 2009). The life cycle assessment (LCA) method has been used to evaluate the environmental trade-offs of P recovery (Bradford-Hartke *et al.*, 2015). LCA results showed that the selection of P recovery method may lead to different environmental benefits and burdens. However, LCA focuses primarily on the environmental impact of emissions, while it overlooks the contribution of ecological products and services. Neglecting the values of nature's work results in incomplete evaluation since natural resources are a significant contributor to all industrial products and processes.

### Diammonium phosphate

The P rocks are enriched in shell fish, bird guano, and bones formed from dissolved P through geological time. Over time, geologic processes bring ocean sediments to land. However, the P returned to the land (by extensive uplifting of sediments) is inadequate to compensate for the loss through erosion, and recently increased intensive agriculture activities (Filippelli, 2011). Treatment of bones with sulfuric acid to increase P mineral (apatite) solubility started in the early to mid-1800s to support increasing agricultural production (Ashley *et al.*, 2011). Today, most of the P fertilizer industrial production is based on acidification of apatite from PR, which has increased significantly for the last 50 years (UNIDO/IFDC, 1998).

Ammonium phosphates, particularly DAP [(NH_4_)_2_HPO_4_], are the most commonly applied fertilizer worldwide and an excellent source of P and N for plant nutrition. The standard grade is 18-46-0 (18% N, 46% P_2_O_5_, and 0% K by weight). The inputs required to produce 1 ton of DAP fertilizer are approximately 1.5–2 tons of PR, 0.4 tons of sulfur to dissolve the rock, and 0.2 tons of ammonia (Shreve and Brink, 1977). The need for recovering ammonia from the crystallizer vapors is avoided by crystallizing DAP from acidic mother liquor (mole ratio ammonium phosphoric acid about 1.6; pH about 6.0). The impurities in the wet-process acid precipitate in a filterable form eventually gelling of the solution (UNIDO/IFDC, 1998).

### Struvite

Struvite recovered from wastewater and urine is a mineral fertilizer with the chemical formula MgNH_4_PO_4_•6H_2_O (magnesium ammonium phosphate hexahydrate). It is formed according to the simplified Equation (1) (Egle *et al.*, 2016). Struvite precipitation is so far one of the most thoroughly researched methods of nutrient recovery with studies spanning the overall effectiveness of the method (Wilsenach, 2006; Liu *et al.*, 2008a, 2008b; Antonini *et al.*, 2011; Etter *et al.*, 2011; Ishii and Boyer, 2015); and the quality of the end product as a fertilizer (Ronteltap *et al.*, 2007; Winker *et al.*, 2009; Decrey *et al.*, 2011).

\begin{align*}
\begin{split}&{ \rm{M}}{{ \rm{g}}^{2 + }} + { \rm{ \;NH}}_4^ + + { \rm{ \;PO}}_4^{3 - } + { \rm{ \;}}6{{ \rm{H}}_2}{ \rm{O}} \\ &\quad\to { \rm{MgN}}{{ \rm{H}}_4}{ \rm{P}}{{ \rm{O}}_4} \bullet 6{{ \rm{H}}_2}{ \rm{O \;}} \left( {{ \rm{solid}}} \right)\end{split}
 \tag{1}
\end{align*}

Struvite has a molar ratio of 1:1:1, NPK-value of ∼6:29:0:(Mg)10 (Barak and Stafford, 2006). It provides slow nutrient release with good P bioavailability activated by the acid released during plant P uptake (Kataki *et al.*, 2016; Talboys *et al.*, 2016). P release in soil appears to be the result of microbial nitrification of the ammonium rather than simple dissolution (Bridger *et al.*, 1962). Struvite formation requires a soluble magnesium source (which often exists in typical municipal wastewater) that leads to possible deposition and clogging of metal appliances and hence entails high cost for the setup of the reactor. The pH control is also critical for optimal struvite precipitation. The pH range at which struvite can precipitate was identified as approximately 7–11, with increasing driving force of precipitation as pH increases (Münch and Barr, 2001; Moss *et al.*, 2013). However, it was argued that precipitates with high struvite content were only obtained at pH 7.0–7.5 (Hao *et al.*, 2013).

Previous life cycle environmental and economic impact studies of source-separated urine and struvite precipitation, when compared with centralized wastewater treatment, have shown that while avoiding fertilizer production does reduce potential impacts, P recovery does not necessarily offset the resources consumed in the process (Ishii and Boyer, 2015). LCA results indicate that selection of an appropriate P recovery method should consider both local conditions and global environmental impacts and mineral depletion impacts (Bradford-Hartke *et al.*, 2015; Ishii and Boyer, 2015). Also, the LCA method does not take into account the ecological products and services, that is, the value (scarcity) of PR. As a thermodynamically based metric, emergy analysis offers a different holistic alternative and biophysical measure that bridges economic and ecological systems. To address the question whether struvite recovery from wastewater is indeed a more efficient strategy from a system perspective and provides more benefits to society, this study compares the full resource use of the fertilizer production of DAP compared with struvite (Crystal Green^®^) using emergy accounting. It quantifies direct and indirect contributions, including PR formation, nitrogen fixation, and the entire supply chains for these two fertilizer production processes. With the unique feature of a single common unit that allows for the comparison of quality difference of all resources (mass, volume, energy, dollar, etc) in system analysis, this study is able to evaluate the emergy expenditure difference between the production of the traditional fertilizer DAP versus struvite generated from domestic wastewater.

## Methodology

### Emergy accounting and theory

Emergy accounting is based on the observation of the energy flow patterns in ecosystems and economic systems during self-organization. Emergy is defined as the available energy required directly and indirectly through all transformations to make a product, process, or service (Odum 1983, 1996). The emergy method provides a quantitative framework to compare different units and scales in a common unit of nonmonetary measure (equivalent solar energy joule, sej). Emergy is a measure of quality differences between different forms of energy. The theory states that the transformation of available energy follows the relative energy quality in a hierarchical order, in which higher transformity values require more upstream energy investment. Such a unique concept of energy quality has the capability to capture the value of natural resources such as rain or P rock that are often considered “free” in the economic market or overlooked in other assessment tools. Emergy theory also emphasizes that prevailing systems are those whose designs maximize available energy by reinforcing resource intake at the optimum efficiency. In other words, the succeeding organization increases intake energy and its efficient use on all scales (not just maximizing levels with more energy, and not maximizing some levels at the expense of others). By holistically assessing all energy, material, and information flows, the behavior of a system as a whole and the interactions between subsystems can be observed and system design can be optimized (Odum, 1996). Nutrient cycling involves food, water, and energy nexus. The complex system requires integrated metrics for a more complete accounting of sustainability and a “common currency” (Hester and Little, 2013). In comparing with other common integrated metrics such as exergy, LCA, or embodied energy, emergy analyses are not only used to evaluate material flows but also services or information. Emergy is a donor-perspective concept, capturing upstream resource use, while other metrics, such as LCA, are user perspective focusing on downstream impacts (Ridolfi and Bastianoni, 2008).

In emergy analysis, the various available energy values from environmental (e.g., rain, energy, and minerals), social (e.g., educational attainment), and economic (e.g., labor) sources are converted into the equivalence of solar emjoules (sej), that is, solar energy memory. Thus, the value of any storage or flow can be assessed inclusively and objectively in terms of its solar emergy (Odum, 1996; Campbell, 2001). A specific quality factor Unit Emergy Value (*UEV_i_*) is used for each input flow of material, energy, or labor (*x_i_*), representing a characteristic emergy value for each pathway. A UEV is expressed in units of sej per mass, volume, energy, or dollars (depending on the particular flow, *x_i_*). They serve as converting factors to transform all materials, products, and services into a system of common emergy units. The quantities of mass, volume, energy, or dollars are expressed in *x_i_*_._ Equation (2) represents the basic emergy accounting formulas used to estimate energy transformation of a process or product.

\begin{align*}
Emergy = \mathop \sum \limits_{i = 1}^{i = n} UE{V_i}^{ \rm{*}}{x_i} \tag{2}
\end{align*}

The special form of UEV is transformity (sej/J; the ratio of emergy input to energy output) and the specific emergy (sej/g; the ratio of emergy input to mass output). Transformity is also an indicator of how much cumulative available energy from the biosphere (natural processes) and technosphere (industrial processes) is required to manufacture a given product. To make the same product, higher transformity of a process requires more overall energy inputs and therefore is less sustainable. More detailed information regarding the energy transformation hierarchy can be found in Supplementary Appendix S1 and Supplementary Fig. S1. This study uses the emergy method to capture the full resource use of products and compares the total emergy inputs with produce 1 ton of DAP and struvite (Crystal Green).

### Emergy evaluation or accounting procedure

**FIG. 1.**
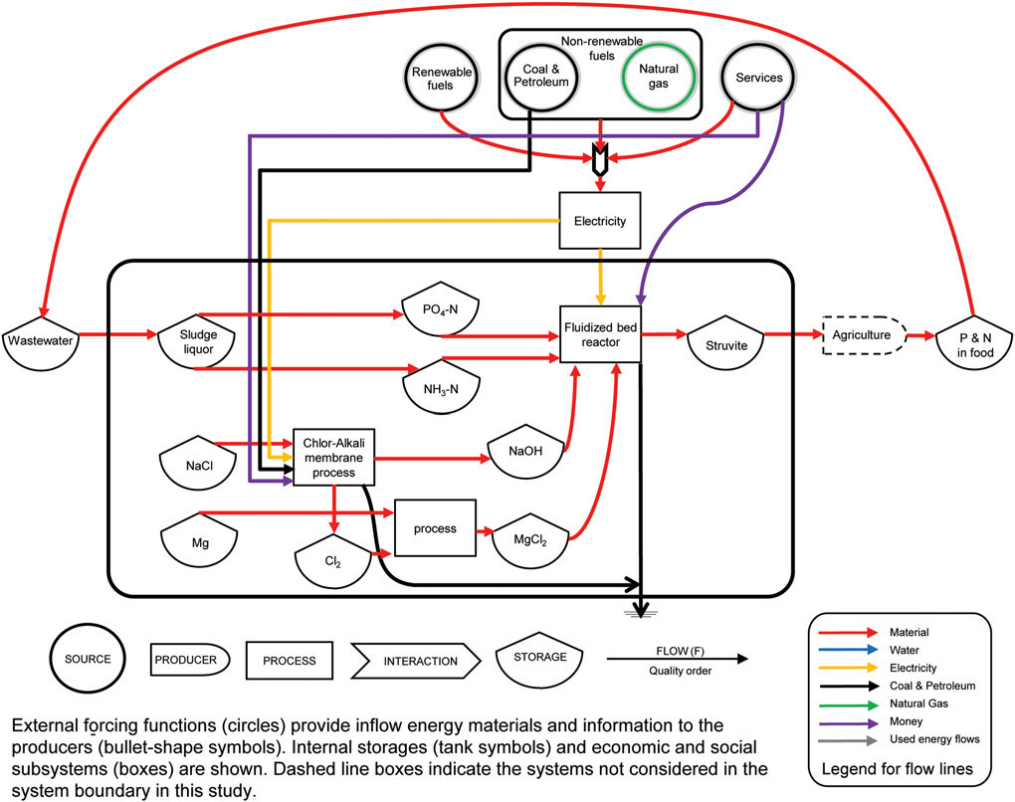
Energy systems diagram showing the formation and extraction of PR to the manufacture of DAP fertilizer. DAP, diammonium phosphate; PR, phosphate rock.

**FIG. 2.**
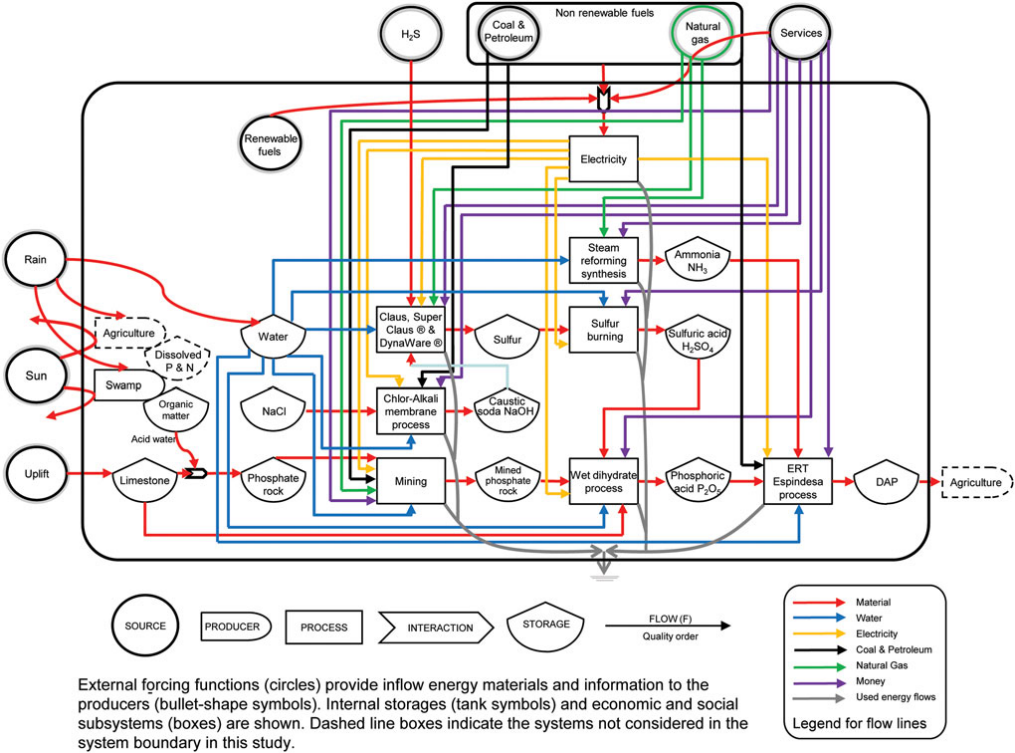
Energy systems diagram showing the process of nutrient recovery and precipitation of struvite (Crystal Green^®^) from secondary treated municipal wastewater.

Emergy accounting begins with energy system diagramming, with the pathways of the diagram determining the line items in an emergy evaluation table. The left-to-right flow direction in the diagram represents the hierarchical order, starting from the left side of global elementary flows such as solar, geothermal, gravitational, tidal, and rain flows toward the right side of the industrial processes (Figs. 1 and 2). Emergy values of each flow are derived from the conversion factors of the UEVs for each product, service, and process (Supplementary Table S1) (Odum, 1996). For the flows of labor and services, emergy contributions are derived from the ratio of the total emergy for the specific country or region to the gross economic product in emdollars (abbreviated em$) (Campbell *et al.*, 2014). Figures 1 and 2 represent the overview system diagrams for DAP manufacture and struvite (Crystal Green) processes, respectively.

Emergy tables provide a tabulation for the emergy values of different storages and flows in Figs. 1 and 2. Raw data on the mass of flows and storage reserves are converted to energy, mass, or dollar and then to emergy by UEVs and emdollars to aid in comparisons. Then total emergy inputs to the system are added up. Renewable and nonrenewable flows are distinguished in the emergy balance sheet. Tables 1 and 2 show the emergy analysis for DAP and struvite (Crystal Green) production, respectively. Tables of the actual flows of materials, labor, and energy, and the hierarchical order are constructed from the diagram (Figs. 1 and 2 and Supplementary Tables S1–S14). Inputs that come from the same source are not added, to avoid double counting, only the larger input is accounted for. For example, in struvite emergy table, emergy flows of phosphate and ammonia are not included because wastewater input is accounted for to avoid double counting. If the table is for the evaluation of a process, it represents flows per unit time (usually per year). If the table is for the evaluation of reserve storages, it includes those storages with a turnover time longer than a year (Odum, 1996).

**Table 1.** Emergy Accounting of Fertilizer Production Processes: Emergy Evaluation of 1 Ton of Diammonium Phosphate Fertilizer

*Note*	*Description*	*Data*	*Unit*	*UEV (sej/unit)*	*Emergy (E sej/y*ea*r)*
Chemical formula: (NH_4_)_2_HPO_4_ composition: 18% N, 46% P_2_O_5_ (20% P)					
Infrastructure input					
	Capital^a^	1.14E+01	$	2.02E+12	2.31E+13
Operational inputs per year (2013)					
1	Materials				
1a	PR	1.50E+06	g	3.61E+09	5.40E+15
1b	Ammonia	1.44E+05	g	6.70E+09	9.67E+14
1c	Sulfur	3.97E+05	g	8.88E+10	3.53E+16
1d	Limestone	3.02E+04	g	2.20E+08	6.65E+12
2	Energy				
2a	Electricity	1.16E+08	J	4.23E+05	7.85E+12
2b	Fuels	4.34E+08	J	9.93E+05	6.12E+13
3	Services	5.12E+02	$	2.02E+12	1.04E+15
4	Water	3.56E+01	m^3^	8.22E+11	1.28E+13
	Total emergy				**4.28E+16**
5	UEV	Without capital invest		**4.28E+10**	sej/g DAP
		With capital invest		**4.28E+10**	sej/g DAP
		With capital invest		**1.01E+10**	sej/g P

The inputs required to produce 1 ton of DAP fertilizer are approximately 1.5–2 tons of PR, 0.4 tons of sulfur (S), to dissolve the rock, and 0.2 tons of ammonia.

Bold values are the total annual emergy inputs for 1 ton DAP and UEVs for DAP and P.

^a^ Material and Capital Costs Data Source: The Mosaic Company (2013).

DAP, diammonium phosphate; N, nitrogen; P, phosphorus; PR, phosphate rock; UEV, unit emergy value.

**Table 2.** Emergy Accounting of Fertilizer Production Processes: Emergy Evaluation of 1 Ton of Struvite (Crystal Green) Using WASSTRIP^a^ and PEARL Processes

*Item*	*Description*	*Data*	*Unit*	*UEV (sej/unit)*	*Emergy (E sej/y*ea*r)*
Chemical formula: Crystal Green, NH_4_MgPO_4_·6H_2_O (5-28-0 + 10% Mg)					
Annual construction input					
	Capital^b^	2.47E+02	$	2.02E+12	5.01E+14
Operational inputs per year (2013)					
1	Materials				
1a	Phosphate, equivalent to elemental phosphorus (PO_4_-P)	1.40E+05	g		0.00E+00
1b	Ammonia, equivalent to elemental nitrogen (NH_3_-N)	2.10E+05	g		0.00E+00
1c	Sodium hydroxide (NaOH)	4.90E+04	g	4.14E+09	2.03E+14
1d	Magnesium chloride (MgCl_2_) as Mg	1.47E+05	g	4.52E+10	6.38E+15
2a	Electricity	6.40E+08	J	2.21E+05	1.41E+14
3	Services	5.33E+01	$	2.02E+12	1.08E+14
4	Wastewater	2.63E+02	g	7.29E+11	1.92E+14
	Total emergy				**7.29E+15**
5	UEV	Without capital invest		**7.29E+09**	sej/g CG
		With capital invest		**7.79E+09**	sej/g CG
		With capital invest		**9.84E+08**	sej/g P

^a^ Source: International Plant Nutrition Institute (2017).

^b^ Material and Capital Costs Data Source: Ostara Nutrient Recovery Technologies, Inc. (2013).

Bold values are the total annual emergy inputs for 1 ton struvite (Crystal Green) and UEVs for Crystal Green and P.

### DAP manufacture

The commercial DAP manufacturing process is an adaptation of the pipe-cross reactor process using wet-process phosphoric acid. Vapor or liquid anhydrous ammonia and phosphoric acid (40–45% P_2_O_5_) are metered continuously to an agitated atmospheric tank (preneutralizer) in proportions to maintain a ratio of 1.3–1.5 mol of ammonia per mole of phosphoric acid. In the preneutralizer, the heat of reaction elevates the temperature of the mass, evaporating ∼200 lb (91 kg) of water per ton of product (International Plant Nutrition Institute, 2017). This is called the ERT-Espindesa process. For granular DAP, most of the ammonia lost from the granulator is recovered in a two-stage ammonia scrubber. The partially neutralized acid from the scrubber has an NH_3_:H_3_PO_4_ molar ratio of about 0.25 and is fed to the pipe reactor where the ratio is raised to 1.9–2.05 (UNIDO/IFDC, 1998). Figure 1 shows the system diagram of DAP manufacture, starting from the formation of the PR using natural or renewable resources, mining and ore refining, and burning elemental sulfur to make sulfuric acid, which later reacts with PR to form phosphoric acid used in the ERT-Espindesa process. The infrastructure, material, energy, services, and water inputs required for DAP manufacture were obtained from The Mosaic Company (2013).

### Struvite recovery

An alternative strategy is extractive nutrient recovery, in which energy and resource are used to accumulate and produce a nutrient product (struvite) that has value in a secondary market (Water Environment Federation, 2013). The extractive nutrient recovery often follows a three-step framework: (1) accumulation of nutrients to high concentrations, (2) release of nutrients to a small liquid flow with low organic matter and solid content, and (3) extraction and recovery of nutrients as a chemical nutrient product. More than 84% of wastewater treatment facilities in the United States use some form of biological accumulation process, in which nutrients, mostly P, are uptaken and stored in biomass (U.S. Environmental Protection Agency, 2012). Release technologies recover the nutrients into a low-flow high-nutrient content stream with minimal solid content, which can be used for extraction processes. This study characterizes the enhanced P release process WASSTRIP^™^ (waste-activated sludge [WAS] stripping to recover internal phosphate) (Baur, 2009) and subsequent PEARL^®^ extraction process (Ostara Nutrient Recovery Technologies, Inc., 2013). This process uses an upward flow fluidized bed reactor with multiple reactive zones of increasing diameters under anaerobic conditions. Subsequent sludge thickening diverts released nutrients into thickening filtrate, which the PEARL process recovers. The WASSTRIP process controls struvite precipitation throughout the sludge treatment stream by reducing the phosphate and magnesium content of the WAS before anaerobic digestion (where ammonia forms). This improves sludge treatment performance, tackles struvite-related maintenance, and significantly reduces sludge production. Struvite production without WASSTRIP and only the PEARL process is also evaluated as a comparison (Supplementary Table S4).

The harvested prills are separated from the treated liquors, dried, screened, and bagged on-site. The marketed fertilizer contains 5% nitrogen, 28% phosphate, and 0% potash, with 16.6% magnesium oxide (10% Mg). The high fluid velocity in the bottom of the reactor also results in the washout of residual sludge solids, producing therefore a purer struvite product, free from organic material and pathogens. The infrastructure, material, energy, services, and water inputs supplied for precipitation of struvite (Crystal Green) were obtained from Ostara Nutrient Recovery Technologies, Inc. (2013). Struvite (Crystal Green) crystallization is controlled by a combination of magnesium dose and pH control, and by means of treated effluent recycle (Ostara Nutrient Recovery Technologies, Inc., 2013). The struvite recovery process is typically integrated into a municipal wastewater treatment in the sludge thickening and dewatering reject water systems. Figure 2 shows the systems diagram for struvite recovery from wastewater.

### Data for emergy accounting

The transformities and UEVs for the raw materials are summarized in Table 1 with details calculated in the Supplementary Data. Some of the UEVs not available in literature were calculated in this study, shown in Supplementary Data. The globe emergy baseline used is 1.2 × 10^25^ sej/year (Brown *et al.*, 2016) and all the UEVs were adjusted to this baseline.

## Results and Discussion

A comparison of the system diagrams of DAP (Fig. 1) and struvite (Crystal Green) (Fig. 2) reveals the complexity of DAP production. Tables 1 and 2 provide the emergy estimates for 1 ton of both DAP manufacture and struvite recovery (Crystal Green), respectively. Supplementary Table S1 provides the list of UEVs used in this study. Supplementary Table S2 shows the input materials, energy, and costs used to calculate the emergy of DAP from the theoretical source (UNIDO/IFDC, 1998), whereas Supplementary Table S3 shows the data and calculations obtained from Agrium, Inc., a U.S. fertilizer manufacturer (Agrium, Inc., 2013). These two production processes showed the potential variation in DAP production process due to energy and water use. Even with the range of DAP production, it is still an order of magnitude higher than struvite production. Supplementary Tables S4–S6 show estimates for proposed struvite (Crystal Green) recovery from both mixed wastewater and source-separated urine. Supplementary Tables S7–S14 provide the UEV calculations for some of the raw chemical inputs (PR formation, PR mining, phosphoric acid, sulfur, sulfuric acid, ammonia, and sodium hydroxide) used in fertilizer recovery or manufacturer processes listed in Supplementary Table S1.

Estimates in Tables 1 and 2 show the UEVs (sej/g of fertilizer or sej/g of P) of the two fertilizers. By definition, the higher the UEV of a product the greater the environmental resources needed to produce it (Brown and Ulgiati, 1997). DAP shows an order of magnitude higher UEV (1.18 × 10^10^ sej/gP) than that of struvite (Crystal Green) with WASSTRIP (9.6 × 10^8^ sej/gP), which is indicative of a bigger environmental emergy “footprint.”

**FIG. 3.**
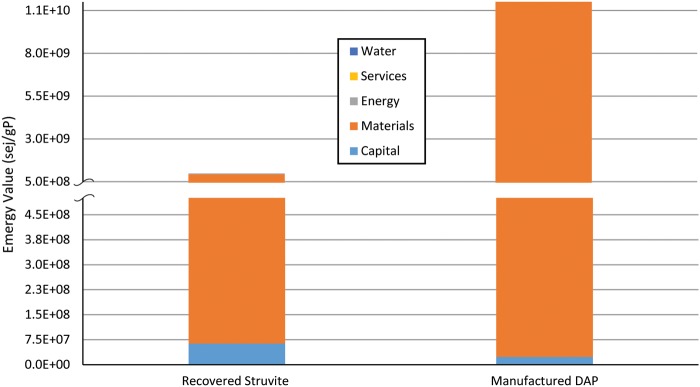
Unit emergy value (sej/gP) comparison for struvite (Crystal Green) recovery and DAP manufacture process.

Figure 3 shows the overall difference and the breakdown of the UEVs for both fertilizers. The DAP process significantly depends on nonrenewable energy sources and a scarce material (PR), whereas struvite taps into the inherent energy present in both N and P wastewater residuals, instead of these residuals being discharged into the environment as pollutants. Otherwise, natural systems (or built engineering systems) must invest substantial emergy to assimilate nutrients, which results in significant loss of important ecosystem services due to the shifting of the structure and organization of these natural systems. In the emergy analyses of both fertilizers, the energy (both direct and indirect) consumed, capital investment, water use, and labor services together contribute ∼3% and 10% to the total UEV (per gram of P available) of DAP and struvite, respectively. The capital investment appears to contribute more to the overall UEV in the struvite recovery because the long-term operation and maintenance contribution portion is less, and therefore, the total emergy is reduced. The detailed analysis reveals that the traditional economic accounting fails to capture the true resource value and the scarcity of P. For example, electricity use is not the dominant emergy contributor in the direct fertilizer process chain, but it is in the production of indirect upstream inputs, P_2_O_5_ (from mining of P to refining of the phosphates), H_2_SO_4_, natural gas, and so on. The chemicals used in the manufacture of both fertilizer supply chains, especially those for DAP production such as nonrenewable elemental sulfur and PR resources with long turnover times, are the highest emergy contributors (>90%) with their higher UEVs and the given quantities applied. The emergy contribution from each chemical is detailed in Supplementary Fig. S1 and Supplementary Tables S10–S14.

The economy of scale in struvite production was investigated by emergy estimation for production of large quantities of struvite at a large wastewater treatment. This estimation was done for the Metropolitan Water Reclamation District of Greater Chicago located at Stickney, IL, with a treatment capacity of 1,200 millions of gallons per day with WASSTRIP and PEARL processes for 10,000 tons Crystal Green production annually. The estimation (shown in Supplementary Table S5) shows that the economy of scale is observed at a larger scale struvite production with less emergy inputs, due to less magnesium chloride use and lower energy consumption. The struvite production was also compared with (Table 2) and without the WASSTRIP (Baur, 2009) process (Supplementary Table S4). It did validate the performance improvement of WASSTRIP (Baur, 2009) because of less chemical addition, less energy used, reduced sludge production, and increased dewaterability of sludge. To better understand if the emergy investments are significant for different sources of struvite, total emergy was calculated in Supplementary Table S6 for source-separated urine (Ishii and Boyer, 2015), which has higher concentrations of P and ammonia. It was clear that due to the lower stability of P in urine, more sodium hydroxide and magnesium chloride are added to maintain the pH of the separated urine and to precipitate out struvite. Although less energy is used, less struvite is generated. Therefore, higher emergy, similar to the PEARL process without WASSTRIP (Supplementary Table S4), is embedded in struvite derived from source-separated urine. Nevertheless, both struvite production methods have significantly lower total emergy values than the one for DAP manufacture.

The results show the promising potential of nutrient recovery from municipal wastewater and provide a sound thermodynamically based comparison of struvite production versus DAP production. Emergy accounting allows the estimation of the hidden ecological costs of producing DAP and struvite and measures the real wealth embodied in these products, counting both environmental services and the technosphere inputs. This donor-side perspective for assessing production has the benefit over economic cost or energy analysis in the ability to value renewable and nonrenewable inputs both from the economy (i.e., labor and services) and from nature (i.e., materials) and to compute their values on a common basis. For example, when the two industrial processes of nitrogen fixation and reduction/removal from wastewater are linked by the direct consumption of minerals in wastewater, the high energy spent to nitrify ammonia, denitrify wastewater nitrates, and fix the nitrogen gas in synthetic fertilizer can be offset by the recycling of ammonia. Similarly, when phosphate production (including mining) and phosphate removal from wastewater are linked, the high energy used in mining, the chemical production spent to precipitate phosphates, and phosphate removal can be offset by the recycling of struvite. Therefore, when the contributions of ecological services are factored in how society functions, real wealth can be reflected in sustainable development.

The observations for this emergy comparison of these two processes align with the emergy principle that sustainable systems are those whose designs maximize available energy by reinforcing resource intake and optimized system efficiency. In this case, struvite recovery captures a previously untapped resource of P and ammonia. Therefore, it shows the lower UEV and demonstrates a much higher system efficiency than the traditional commercial fertilizer. The emergy accounting method demonstrated a unique quantification of biophysical-based sustainable production of struvite.

Although the objective of the study is to compare the resource inputs for 1 ton of fertilizer produced, not all benefits and impacts are captured in the current analysis. For example, significant credits, such as avoiding nutrient removal treatment processes mandated for wastewater treatment plants to meet the permitted total maximum daily loads or NPDES regulations, have not been accounted for. The direct financial benefit is the new revenue stream of fertilizer production (Shu *et al.*, 2006; Kleemann, 2015). A further comparison of nutrient removal versus nutrient recovery may shed light on how to best manage nutrients from a system perspective and provide more regulatory insights.

In addition, the indirect benefits can be fully evident when the system boundary is expanded to include the performance, uptake, and loss during the fertilizer use phase. The inherent slow release nature of struvite implies that the amount required for the same level of crop production will be less, leading to less runoff (Talboys *et al.*, 2016). Expanding the system boundary helps understand all aspects of the entire nutrient cycle. The system efficiency can be further revealed by comparing both fertilizers in terms of crop production, nutrient uptake, nutrient runoff or erosion, and eventual release in the waste stream. The system-level analysis will provide a more complete evaluation of nutrient recovery, the implication for long-term sustainable agriculture production, and the reduction of environmental impacts such as avoided mining of nonrenewable resources and avoided eutrophication and energy use.

## Conclusions

This study evaluates the emergy requirements of struvite-based fertilizer and the commercial fertilizer DAP production from “cradle to gate” (resource use to the factory gate). The results of the emergy accounting support the premise that nutrient recovery from municipal wastewater is a promising sustainable alternative in the water-nutrient-food nexus system management. When the total resource use (natural and economic resources) is quantified, the struvite (Crystal Green) recovery process shows less emergy requirement by capturing untapped resources, maximizing resource use, and optimizing the overall system efficiency. Emergy analysis shows almost one order of magnitude higher specific emergy in 1 unit of P presented in DAP than that of struvite due to the more intense use of nonrenewable resources and the high emergy embedded chemicals used in DAP production. Future research should focus on expanding the overall understanding of the benefits of nutrient recovery (N and P) when resource recovered fertilizers are compared with commercial fertilizers in field application. Further study beyond the fertilizer production phase is needed to have more complete comparisons between struvite (slow-release, low solubility in water, cost driven by the bioavailable P) and DAP (instantaneous-release, highly soluble, and higher P content) in the context of nutrient cycling. With a system perspective, thermodynamic-based analysis can provide the evidence needed to demonstrate the economic and environmental benefits to the society of nutrient recovery. The results shed some light on holistic sustainable management of resource use, nutrient management, water quality, food security, and energy consumption to achieve overall system efficiency.

## Supplementary Material

Supplemental data
