# Supplementary material for: Nutrient Recovery from Municipal Wastewater for Sustainable Food Production Systems: An Alternative to Traditional Fertilizers
Source: Environ Eng Sci. 2019 Jul 10;36(7):833–42. doi: 10.1089/ees.2019.0053 (PMC6653797; doi:10.1089/ees.2019.0053)
Supplement: Supplemental data [file Supp_Data.pdf]

Supplementary Data

Supplementary Appendix 1
Unit Energy Values and Emergy Estimations of Inputs Used for Struvite Recovery and Diammonium Phosphate Manufacture

Emergy Accounting Definitions

Unit emergy values (UEVs) are based on the emergy required to produce something. UEVs are calculated by dividing the sum of all emergy required by the units of product output. There are two types of UEVs appropriate for this chapter as follows: Transformity is defined as the emergy per unit of available energy (exergy). For example, if 4,000 solar emjoules are required to generate a joule of wood, then the solar transformity of that wood is 4,000 solar emjoules per joule (abbreviated sej/J). Solar energy is the largest but most dispersed energy input to the earth. The solar transformity of the sunlight absorbed by the earth is 1.0 by definition (Odum, 1996).

Specific emergy is the UEV of matter defined as the emergy per mass, usually expressed as solar emergy per gram (sej/g). Solids may be evaluated best with data on emergy per unit mass for its concentration. Because energy is required to concentrate materials, the UEV of any substance increases with concentration. Elements and compounds not abundant in nature therefore have higher emergy/mass ratios when found in concentrated form since more work was required to concentrate them, both spatially and chemically (Odum, 1996).

Emergy per unit money is a UEV used to convert money payments into emergy units. The amount of resources that money buys depends on the amount of emergy supporting the economy and the amount of money circulating. An average emergy/money ratio in solar emjoules/$ can be calculated by dividing the total emergy use of a state or nation by its gross economic product. It varies by country and has been shown to decrease each year. This emergy/money ratio is useful for evaluating service inputs given in money units where an average wage rate is appropriate (Odum, 1996).

Emergy accompanying a flow of something (energy, matter, information, etc.) is calculated using a UEV. The flow expressed in its usual units is multiplied by the emergy per unit of that energy or material. For example, the flow of a fuel input to a process, in joules per time, can be multiplied by the transformity of that fuel (emergy per unit energy in solar emjoules/joule), or the mass of a material input can be multiplied by its specific emergy (emergy per unit mass in solar emjoules/gram). The emergy of a storage is calculated by multiplying the storage quantity in its usual units by its UEV (Odum, 1996).

The bar graphs in Supplementary Fig. S1 visualize the energy transformation hierarchy theory, that is, transformity being the inverse of energy transfer/flow in a system. If the successive transformities occur, the energy retained may decrease by a constant percentage with each stage of transformation, as seen in Supplementary Fig. S1a (Odum, 1996). The total emergy indicated as the dash bars stays the same. Therefore, the transformities, as the ratio of the total emergy to the energy in the products, increase along each transformation, indicating the increased embodied energy through each stage, as shown in Supplementary Fig. S1b.

For emergy tables, most commonly, they are set up in a six-column format, in which:

- Column No. 1 is the line item number, and is also the number corresponding to the raw data sources' reference and UEV calculations shown in the Excel spreadsheet.
- Column No. 2 is the name of the item, which is also shown on the aggregated diagram.
- Column No. 3 is the raw data in joules, grams, dollars, or other units.
- Column No. 4 shows the units for each raw data item in Column Nos. 2 and 3.
- Column No. 5 is the transformity used for calculations, expressed in solar emergy joules per joule. Inputs are expressed in different units such as grams, hours, dollars, which are converted to emergy units with the appropriate conversion ratio (sej/hour; sej/g; sej/$). These transformities may be obtained from previous studies or calculated for the system under investigation. If transformities from other studies are used, source reference should be shown in footnotes.
- Column No. 6 is the solar emergy of a given flow, calculated as raw input times the transformity (Column 3 times Column 5).

Supplementary Tables S1–S14 are the solar emergy, UEV, and transformity estimations made for use in the corresponding article. Supplementary Tables S2 and S3 are emergy results for the industrial process data from Chemical Process Industries (Shreve and Brink, 1977) text and Agrium, Inc. (2013), respectively. Supplementary Tables S4–S6 are the emergy estimation for the recovery of struvite (Crystal Green®) using the data from Ostara Nutrient Recovery

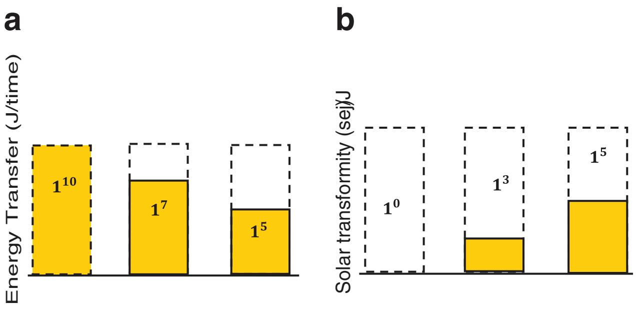

SUPPLEMENTARY FIG. S1. Bar graph representing the concept of energy transformation hierarchy; **(a)** bar graph of the energy flow for the levels of energy hierarchy and **(b)** bar graph of solar transformities (Odum, 1996).

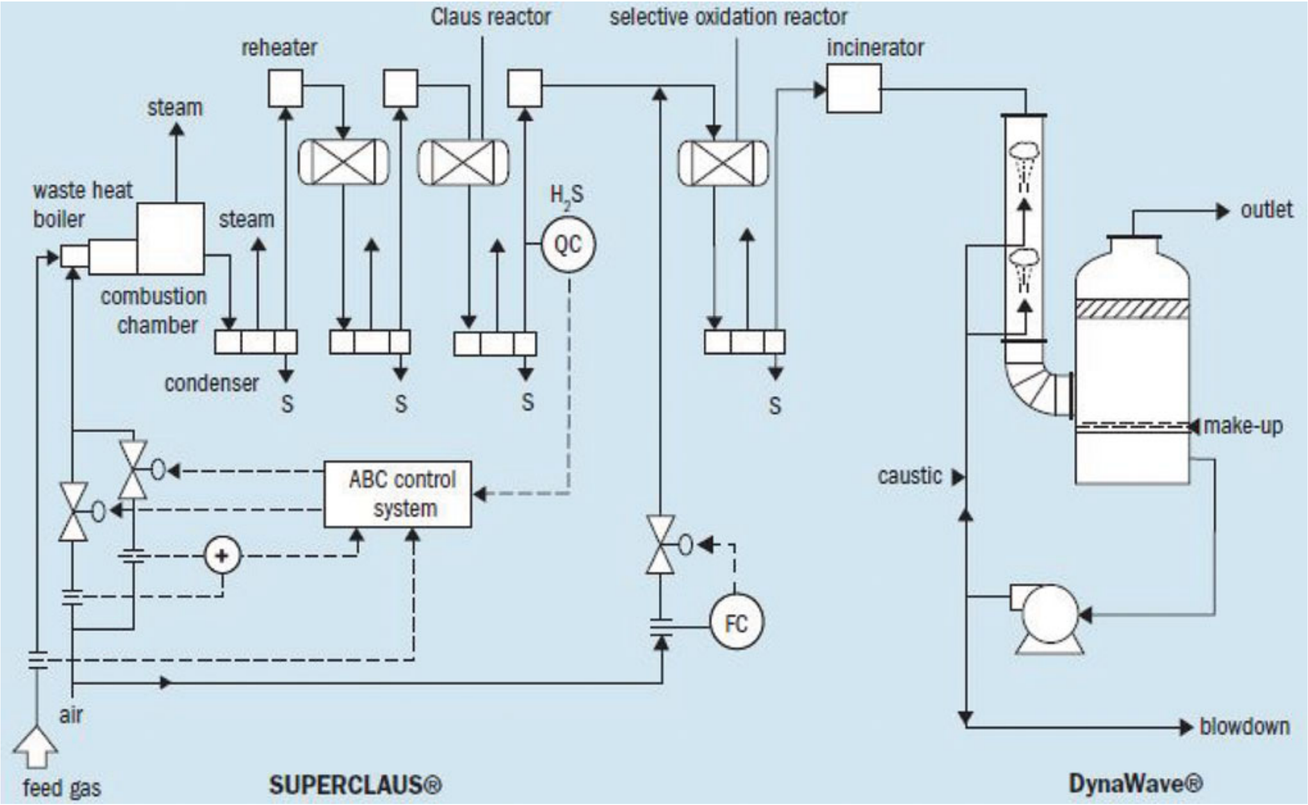

SUPPLEMENTARY FIG. S2. Representation of the two-stage Claus unit with SuperClaus® and Dynawave® for sulfur production.

Technologies, Inc. (2013) and from separated urine, respectively. Supplementary Tables S7, and S9–S14 provide the UEV calculations for phosphate rock (PR), phosphoric acid, elemental sulfur, sulfuric acid, ammonia, and caustic soda (sodium hydroxide), respectively.

Given below, in Supplementary Table S1, is the list of UEVs used for actual estimations shown in the results of the article.

Manufacture of Diammonium Phosphate

Emergy estimations for diammonium phosphate (DAP) manufacture using theoretical data obtained in Shreve and Brink (1977) and industrial data from Agrium, Inc. (2013) are provided in Supplementary Tables S2 and S3. The basic procedure used to manufacture DAP, as explained in the text, is summarized below.

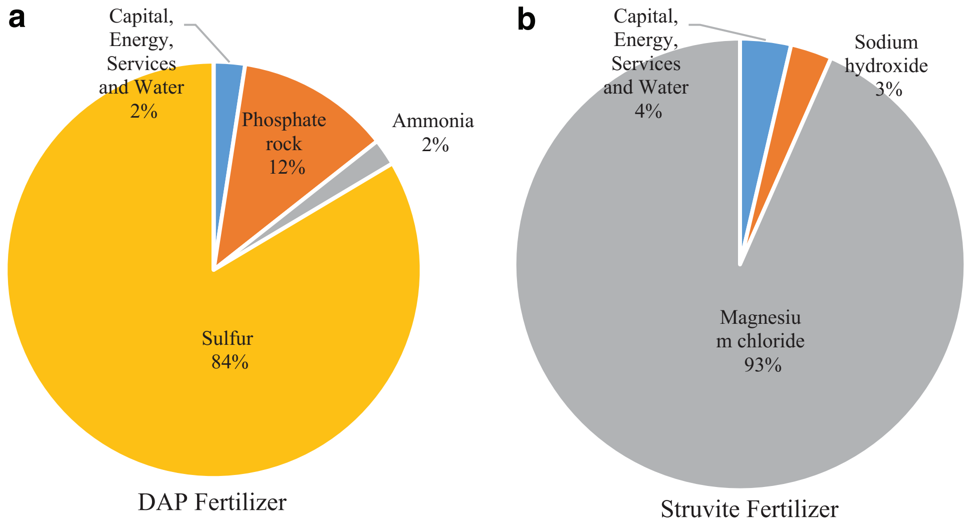

SUPPLEMENTARY FIG. S3. Pie-chart representation of the major emergy contributors in the production process—chemicals added during production of **(a)** DAP and **(b)** struvite. DAP, diammonium phosphate.

Supplementary Table S1. Unit Energy Values and Transformities for Inputs Used in the Struvite and Diammonium Phosphate Production Process

Flow items and commodity class	*Transformity (sej/J)*	*UEV (sej/g)*	*Adjusted transformity (sej/J)*	*Adjusted transformity UEV (sej/g)*
Renewable inflows				
Solar energy reaching the surface of the earth by definition (Odum, 1996)	1		1	
Rainwater converging to a swamp (wetland 2,000 ppm TDS) (Odum, 2000)			3.65E+04	5.22E+06
Fresh groundwater (335 ppm TDS) (Odum, 2000)			1.06E+05	4.96E+05
Fresh groundwater (sej/m^3) (Odum, 2000)				4.96E+11
Fresh surface water, rivers and streams (100 ppm TDS) (Odum, 2000)			2.13E+04	3.26E+05
Fresh surface water (sej/m^3) (Odum, 2000)				3.26E+11
Treated water, potable (sej/m^3) (Arden *et al.*, 2019)				8.61E+11
Wastewater treatment (sej/m^3) (Arden *et al.*, 2019)				7.29E+11
Nonrenewable flows				
Minerals				
Limestone marl uplift[a]				2.23E+10
Limestone (calcite) (McClanahan, 1990)				2.20E+08
Phosphate rock (crude ore)[b]			2.09E+07	3.61E+09
Mined phosphate rock (P content)[b]			3.03E+07	2.13E+10
Mined phosphate rock (P_2O_5 content)[b]				4.68E+09
Magnesium $MgCl_2$ (Cherubini *et al.*, 2008)		5.72E+10		4.52E+10
Potash ore (sylvite) (Vallero *et al.*, 2012)				8.54E+07
Micronutrients				
Boron		4.13E+09		5.35E+09
Zinc (Cohen *et al.*, 2007)		4.11E+10		5.33E+10
Cupric oxide (Cohen *et al.*, 2007)		5.73E+10		7.43E+10
Products				
Phosphoric acid (P_2O_5 content)[a]			9.17E+08	1.02E+11
Elemental sulfur (Koscienlnuk *et al.*, 2010)				8.88E+10
Sulfuric acid, H_2SO_4 (100%) (UNIDO/IFDC, 1998)[a]				3.07E+10
Ammonia (NH_3) (Shreve and Brink, 1977)				6.70E+09
Caustic soda (NaOH) (Campbell and Ohrt, 2009)				4.14E+09
Salt (NaCl) (Hau and Bakshi, 2005)		6.55E+08		8.33E+08
DAP[c]				5.03E+10
DAP (P content)[c]				1.18E+10
DAP (N content)[c]				1.07E+10
Struvite[d]				7.10E+09
Struvite (P content)[d]				9.60E+08
Struvite (N content)[d]				4.33E+08
Fertilizer (DAP or MAP)[e]				4.28E+10
Fertilizer (DAP or MAP) P content[e]				1.00E+10
Fertilizer (DAP or MAP) N content[e]				9.08E+09
Energy				
Electricity (including renewable and nonrenewable sources) (Odum, 1996)	1.74E+05		2.21E+05	
Hydroelectricity (Odum, 1996)	1.23E+05		1.56E+05	
Electricity generated from biogas (Ciotola *et al.*, 2011)	1.01E+06		1.28E+06	
Electricity generated from natural gas (Cohen *et al.*, 2007)	1.53E+05		1.16E+05	
Coal power plant (Odum, 1996)	1.60E+05		2.03E+05	
Industrial steam (Bastianoni *et al.*, 2009)		1.30E+09		1.68E+09
Steam boiler (Caruso *et al.*, 2001)	1.57E+05		2.00E+05	
Cogeneration steam boiler (Caruso *et al.*, 2001)	8.10E+04		1.03E+05	
Nuclear (Campbell and Ohrt, 2009)	4.81E+04		6.23E+04	
Hydropower (Brown and Ulgiati, 2002)	5.76E+04		7.46E+04	
Wind (Brown and Ulgiati, 2002)	5.78E+04		7.49E+04	
Biomass (Odum, 1996)	4.02E+04		5.21E+04	

(*continued*)

Supplementary Table S1. (Continued)

Flow items and commodity class	*Transformity (sej/J)*	*UEV (sej/g)*	*Adjusted transformity (sej/J)*	*Adjusted transformity UEV (sej/g)*
Geothermal (Odum, 1996)	3.37E+04		4.37E+04	
Solar (Photovoltaic Italy) (Paoli *et al.*, 2008)	8.92E+04		1.16E+05	
Average renewable			7.22E+04	
Fuels				
Petroleum derivatives (Bastianoni *et al.*, 2009)	6.58E+04		8.53E+04	
Natural gas (Brown *et al.*, 2011)			1.40E+05	1.46E+11
Natural gas (sej/m^3) (Odum, 1996; Brown *et al.*, 2011)				5.37E+12
Crude oil (Brown *et al.*, 2011)			1.32E+05	1.32E+05
Coal (anthracite) (Brown *et al.*, 2011, 2016)			2.95E+04	9.59E+09
Average fossil fuels			1.01E+05	
Emergy money ratio	Total emergy (sej/year)	gross domestic product, nominal $	Adjusted emergy (sej/year)	Emergy/ money
2011 Value assumed for 2013 (U.S. economy) (Campbell *et al.*, 2014)	2.35E+25	1.51E+13	3.05E+25	2.02E+12

[a]Calculated using literature energy and/or emergy values.
[b]Supplementary Table S7 below.
[c]Supplementary Table S2 below.
[d]Supplementary Table S2 in the article.
[e]Table 1 in the article.
DAP, diammonium phosphate; MAP, monoammonium phosphate; N, nitrogen; P, phosphorus; TDS, total dissolved solids; UEV, unit emergy value.

Granular high-analysis DAP fertilizer

This is basically a commercial adaptation of the (double conical mixer) TVA process, designed for the manufacture of DAP, with wet-process phosphoric acid, or for any of the many grades of fertilizer. Vapor or liquid anhydrous ammonia and phosphoric acid (40–45%) are metered continuously to an agitated atmospheric tank (preneutralizer) in proportions to maintain a ratio of 1.3–1.5 mol of ammonia per mole of phosphoric acid. In the preneutralizer the heat of reaction elevates the temperature of the mass, evaporating ~200 lb of water/ton of product. The slurry formed in the preneutralizer flows into a TVA-type ammoniator-granulator at about 250 F, where it is distributed evenly over the bed of solid material and, for most grades, reacts with additional ammonia fed through a distributor pipe below the surface of the bed to complete the reaction with a molar ratio of 2.0 (DAP). The moist granules leaving the ammoniator fall into an oil- or gas-fired concurrent rotary dryer, where the moisture content of the material is reduced to about 1%. The dry product is then cooled by a countercurrent flow of air in a rotary cooler and screened, the coarse material being milled and returned to the ammoniator-granulator.

Supplementary Table S2. Emergy Evaluation of 1 Ton of Diammonium Phosphate Fertilizer[a]

Note	*Description*	*Data*	*Unit*	*UEV (sej/unit)*	*Emergy (E sej/year)*
Chemical formula: $(NH_4)_2HPO_4$ composition: 18% N, 46% P_2O_5 (20% P)					
Infrastructure input					
	Capital	2.75E+00	$	2.02E+12	5.57E+12
Operational inputs per year (2013)					
1	Materials				
1a	Phosphoric acid (P_2O_5)	4.67E+05	g	9.84E+10	4.60E+16
1b	Ammonia	2.22E+05	g	6.70E+09	1.49E+15
2	Energy				
2a	Electricity	2.59E+08	J	2.21E+05	5.72E+13
2b	Fuels	3.80E+08	J	8.53E+04	3.24E+13
3	Services	6.38E+02	$	2.02E+12	1.29E+15
4	Water	5.00E+04	g	3.26E+05	1.63E+10
	Total emergy				**4.88E+16**
5	Transformity	Without capital invest		**4.88E+10**	sej/g DAP
		With capital invest		**4.88E+10**	sej/g DAP
6	Yield $(NH_4)_2(HPO_4)$	With capital invest		**1.15E+10**	sej/g P
		With capital invest		**1.04E+10**	sej/g N

[a]Material and Capital Costs Data Source: Shreve and Brink (1977).
Bold values are the total annual emergy inputs for 1 ton DAP and UEVs for DAP, N in DAP and P in DAP.

Supplementary Table S3. Emergy Evaluation of 1 Ton of Diammonium Phosphate Fertilizer[a]

Item	*Description*	*Data*	*Unit*	*UEV (sej/unit)*	*Emergy (E sej/year)*
Chemical formula: $(NH_4)_2HPO_4$ composition: 18% N, 46% P_2O_5 (20% P)					
Annual construction input					
	Capital	2.75E+05	\$/year	2.02E+12	5.57E12
Operational inputs per year (2013)					
1	Materials				
1a	Phosphoric acid (P_2O_5)	4.67E+05	g	9.84E+10	4.60E+16
1b	Ammonia	2.22E+05	g	6.70E+09	1.49E+15
2	Energy				
2a	Electricity	1.18E+10	J	2.21E+05	2.61E+15
2b	Fuel	1.12E+10	J	8.53E+04	9.56E+14
3	Services	6.38E+02	\$	2.02E+12	1.29E+15
4	Water	2.30E+00	m^3		8.08E+11
4a	Groundwater	3.45E-01	m^3	4.96E+11	1.71E+11
4b	Surface water	1.96E+00	m^3	3.26E+11	6.37E+11
	Total emergy				**5.23E+16**
	Transformity	Without capital invest		**5.23E+10**	sej/g DAP
		With capital invest		**5.23E+10**	sej/g DAP
	Yield $(NH_4)_2(HPO_4)$	With capital invest		**1.23E+10**	sej/g P
		With capital invest		**1.11E+10**	sej/g N

[a]Material and Capital Costs Data Source: Agrium, Inc. (2013).
Bold values are the total annual emergy inputs for 1 ton DAP and UEVs for DAP, N in DAP and P in DAP.

Struvite Recovery in Large Scale

Phosphorus rock mining and emergy values

PR resource. The world and U.S. natural source of PR is finite and there has been a continuous decrease in world PR quality as reserves of high-quality rock are being depleted (McClellan and Gremillion, 1980). There are two main types of PR deposits—sedimentary and igneous. Sedimentary PRs were formed in continental shelf marine environments, and are thus taken from present or former continental margins. Igneous PR was formed mostly in shield areas and rift zones. Most (>80%) of the PR used in fertilizer production is obtained from sedimentary deposits, but igneous deposits are also used.

PR mining. A wide variety of techniques and many types of equipment are used to mine and process PR. It is mined by both surface (open cast or strip mining) and underground methods. Surface mining is by far the most popular method and most PR is extracted through open pit mining techniques

Supplementary Table S4. Emergy Evaluation of 1 Ton of Struvite (Crystal Green): Without WASSTRIP and only PEARL Process[a] for a 2K Ton Production Annually[b]

Note	*Description*	*Data*	*Unit*	*UEV (sej/unit)*	*Emergy (E sej/year)*
Crystal Green®, $NH_4MgPO_4 \cdot 6H_2O$ (5-28-0 + 10% Mg)					
Annual construction input					
	Capital	1.52E+02	\$	2.02E+12	3.08E+14
Operational inputs per year (2013)					
1	Materials				
1a	Phosphate, equivalent to elemental phosphorus (PO_4-P)	1.40E+05	g		0.00E+00
1b	Ammonia, equivalent to elemental nitrogen (NH_3-N)	4.20E+05	g		0.00E+00
1c	Sodium hydroxide (NaOH)	0.00E+05	g	4.14E+09	0.00E+00
1d	Magnesium chloride ($MgCl_2$) as Mg	4.89E+05	g	4.52E+10	2.21E+16
2	Electricity	1.08E+09	J	2.21E+05	2.38E+14
3	Services	4.26E+01	\$	2.02E+12	8.63E+13
4	Wastewater	5.26E+02	g	7.29E+05	1.71E+08
	Total emergy				**2.24E+16**
	Transformity	Without capital invest		**2.24E+10**	sej/g CG
		With capital invest		**2.27E+10**	sej/g CG
	Yield $(NH_4)_2(HPO_4)$	With capital invest		**2.83E+09**	sej/g P

[a]Source: Ostara Nutrient Recovery Technologies, Inc. (2013).
[b]Material and Capital Cost Data Source: Ostara Nutrient Recovery Technologies, Inc.: personal communication.
Bold values are the total annual emergy inputs for 1 ton struvite and UEVs for Crystal Green and P in Crystal Green.

Supplementary Table S5. Emergy Evaluation of 1 Ton of Struvite (Crystal Green): WASSTRIP[a] and PEARL Processes[b] for 10K Ton Production Annually[c]

Note	*Description*	*Data*	*Unit*	*UEV (sej/unit)*	*Emergy (E sej/year)*
Crystal Green, $NH_4MgPO_4 \cdot 6H_2O$ (5-28-0 + 10% Mg)					
Annual construction input					
	Capital	3.81E+02	$	2.02E+12	7.70E+14
Operational inputs per year (2013)					
1	Materials				
1a	Phosphate, equivalent to elemental phosphorus (PO_4-P)	1.52E+05	g		0.00E+00
1b	Ammonia, equivalent to elemental nitrogen (NH_3-N)	5.91E+05	g		0.00E+00
1c	Sodium hydroxide (NaOH)	3.55E+05	g	4.14E+09	1.82E+15
1d	Magnesium chloride ($MgCl_2$) as Mg	4.03E+05	g	4.52E+10	1.14E+16
2	Electricity	1.00E+09	J	2.21E+05	1.28E+14
3	Services	1.07E+02	$	2.02E+12	4.31E+14
4	Wastewater	1.92E+02	g	7.29E+05	3.46E+10
	Total emergy				**2.01E+16**
	Transformity	Without capital invest		**2.01E+10**	sej/g CG
		With capital invest		**2.09E+10**	sej/g CG
	Yield $(NH_4)_2(HPO_4)$	With capital invest		**2.54E+09**	sej/g P

[a]Source: Baur (2009).
[b]Source: Ostara Nutrient Recovery Technologies, Inc. (2013).
[c]Source: Material and Capital Cost Data Source: Ostara Nutrient Recovery Technologies, Inc.: personal communication.
Bold values are the total annual emergy inputs for 1 ton struvite and UEVs for Crystal Green and P in Crystal Green.

Supplementary Table S6. Emergy Evaluation of 1 Ton of Struvite (Crystal Green): WASSTRIP[a] and PEARL Processes[b] from Stored Urine (250,000 gpd)[c]

Note	*Description*	*Data*	*Unit*	*UEV (sej/unit)*	*Emergy (E sej/year)*
Crystal Green, $NH_4MgPO_4 \cdot 6H_2O$ (5-28-0 + 10% Mg)					
Annual construction input					
	Capital	1.52E+02	$	2.02E+12	3.08E+14
Operational inputs per year (2013)					
1	Materials				
1a	Phosphate, equivalent to elemental phosphorus (PO_4-P)	1.47E+05	g		0.00E+00
1b	Ammonia, equivalent to elemental nitrogen (NH_3-N)	1.98E+06	g		0.00E+00
1c	Sodium hydroxide (NaOH)	2.80E+05	g	4.14E+09	1.16E+15
1d	Magnesium chloride ($MgCl_2$) as Mg	5.32E+05	g	4.52E+10	2.40E+16
2	Electricity	6.56E+08	J	2.21E+05	1.45E+14
3	Services	4.26E+01	$	2.02E+12	8.63E+13
4	Wastewater	5.07E+02	g	7.27E+5	3.7E+08
	Total emergy				**2.54E+16**
	Transformity	Without capital invest		**2.54E+10**	sej/g CG
		With capital invest		**2.57E+10**	sej/g CG
	Yield $(NH_4)_2(HPO_4)$	With capital invest		**3.21E+09**	sej/g P

[a]Source: Baur (2009).
[b]Source: Ostara Nutrient Recovery Technologies, Inc. (2013).
[c]Urine data source: Ishii (2015).
Bold values are the total annual emergy inputs for 1 ton struvite and UEVs for Crystal Green and P in Crystal Green.

Supplementary Table S7. Emergy Evaluation of Phosphate Rock Formation[a]

Item	*Description*	*Data*	*Unit*	*UEV (sej/unit)*	*Emergy (sej/year)*
Inputs					
1	Sun	2.78E+14	J	1	
2	Limestone marl uplift	2.24E+06	g	2.04E+08	4.57E+14
3	Rain	2.93E+10	J	3.65E+04	4.00E+15
4	Total emergy				4.45E+15
5	Gibbs free energy for P	3.66E+02	J/g		
6	Total energy in 200 kg rock[a]	7.32E+07	J		
	UEV for phosphate rock			**2.23E+10**	sej/g rock

[a]200 kg 10% phosphate rock after its concentration from 2,000 kg limestone marl (from 10 m^2 area) by percolation of organic-rich swamp waters (into 1 m^2).
Bold value is the UEV for phosphate rock.
Revised from Odum (1996), p. 124, Table 7.7.

Supplementary Table S8. Emergy Evaluation of 1 Ton of Mined Phosphate Rock

Item	*Description*	*Data*	*Unit*	*UEV (sej/unit)*	*Emergy (sej/year)*
1	Phosphate rock crude ore	3.00E+06	g	3.61E+09	1.08E+16
2	Energy	1.56E+08	J		3.04E+13
2a	Electricity	1.22E+08	J	2.21E+05	2.70E+13
2b	Fuels	2.44E+07	J	8.53E+04	2.08E+12
2c	Natural gas	8.89E+06	J	1.40E+05	1.30E+12
3	Services	1.02E+02	\$	2.02E+12	2.07E+14
4	Water	3.42E+00	m^3		1.52E+12
4a	Groundwater	2.39E+00	m^3	4.96E+11	1.19E+12
4b	Surface water	1.03E+00	m^3	3.26E+11	3.37E+11
	Total emergy				**1.11E+16**
	UEV for phosphate mined rock	Without capital invest		**1.11E+10**	sej/g Phosphate
				**5.07E+10**	sej/g P

Bold values are the total annual emergy inputs for 1 ton mined phosphate rock and UEVs for phosphate and P.
Source: Odum (1996).

such as small diesel draglines, bucket wheel excavators, and front end loader removal.

Beneficiation of PR. Concentrated (beneficiated) PRs are usually about 27–37% P_2O_5 (may be as low as ~23%) (UNIDO/IFDC, 1998). Initial removal of impurities from PR ore is called beneficiation. Beneficiation of PR involves removal of materials such as sand, clay, carbonates, organics, and iron oxide. Beneficiation may involve screening (wet or dry), washing, hydrocyclones, calcination, flotation, and use of magnets. Ore washing and screening separate oversize material (3–20 cm), and remove clays and other fines, which result in a slurry of suspended waste called "slime." In areas without sufficient water, dry screening may be used. Froth flotation requires deslimed feedstock and the first step involves bubbling air through an anionic collector such as fatty acid. Fine ore is passed through flotation cells, and PR is attracted to the anionic collector and rises with froth. Floating apatite is thus separated from silica tailings by overflow or paddlewheels. Ore calcination is removal of carbonates that are in the organic form by passing the ore through the furnace at high temperatures, mainly to improve the quality of the final product, and is used mainly where the cost of natural gas is low.

Conversion of PR to phosphoric acid. After beneficiation, PR is converted to phosphoric acid, which is mainly used in phosphate fertilizers. The two basic processes of phosphoric acid production are wet (chemical) process and electric furnace (thermal) process. The furnace process is used extensively for nonfertilizer purposes and is unlikely to be competitive for fertilizer production. The majority of phosphorus (P) fertilizer is produced by wet process, which

Supplementary Table S9. Emergy Evaluation of Phosphate Formation and Mining Florida: Forming and Mining[a]

Item	*Description*	*Data, units*	*Transformity sej/unit*	*Emergy E+12 sej*
Calculations corrected and with updated assumptions and numbers. Table 7.7 Emergy evaluation of phosphate formation and mining Florida: Forming and mining 200 kg 10% rock after its concentration from 2,000 kg original limestone marl by percolation of organic-rich swamp waters[b]				
Phosphate rock formed, 200 kg				
1	Direct sun, 10 m^2 of land	2.73E+14 J		273
2	Limestone marl uplift	2.24E+06 g	1.42E+08	320
3	Rain and runoff	2.22E+10 J	1.81E+04	402
4	Total solar emergy into rock (2 + 3)			722
Mined rock phosphate, 180 kg				
5	Service	\$2.84	7.06E+12	20.1
6	Fuel	1.58E+08J	6.58E+04	10.4
7	Electricity	6.99E+08J	1.30E+05	91
8	Total solar emergy in mined phosphate rock			843
	Solar emergy per unit calculated by dividing total solar emergy (previously used by the emergy or weight, respectively)			
	Phosphate rock in the ground		**3.61E+09**	sej/g $CaPO_4$
	Mined rock		**4.68E+09**	sej/g $CaPO_4$
			**2.13E+10**	sej/g P

[a]Erratta Odum (1996), pp. 124–125. Calculations corrected and with updated assumptions and numbers.
[b]Time for development of phosphate deposit estimated from rate of percolation through Florida swamp: 10% of rain over 10 m^2 runs into swamp area of 1 m^2; 2.5 cm/week (130 cm/year) percolated down carrying 100 mg/L organic matter that oxidizes, generating acid as it percolates, dissolving limestone.
Bold values are different emergy sums (UEVs) for $CaPO_4$ and P.

Supplementary Table S10. Emergy Evaluation of H_3PO_4 (32% P_2O_5 in Acid per Ton)[a]

Note	Description	Data	Unit	UEV (sej/unit)	Emergy (sej/year)
Annual construction input					
	Capital	9.43E+00	\$	2.02E+12	1.91E+13
Operational inputs per year (2013)					
1	Mined P rock	3.29E+06	g	4.68E+09	1.54E+16
2	Electricity	3.60E+08	J	2.21E+05	7.95E+13
3	Services	4.08E+02	\$	2.02E+12	8.27E+14
4	Water	7.89E+01	m^3		
4a	Groundwater	5.51E+01	m^3	4.96E+11	2.73E+13
4b	Surface water	2.38E+01	m^3	3.26E+11	7.76E+12
5	H_2SO_4 (100%)	2.78E+06	g	3.27E+10	8.53E+16
6	Limestone	1.50E+05	g	2.20E+08	3.31E+13
	Total emergy				**9.84E+16**
	UEV for P_2O_5	Without capital invest		**9.84E+10**	sej/g P_2O_5
				**4.51E+11**	sej/g P

[a]Source: UNIDO/IFDC (1998).
Bold values are the total annual emergy inputs for 1 ton P_2O_5 and UEVs for P_2O_5 and P.

Supplementary Table S11. Emergy Evaluation of 1 Ton of Recovered Sulfur

Note	Description	Data	Unit	UEV (sej/unit)	Emergy (sej/year)
Operational inputs					
1	Hydrogen sulfide gas (H_2S)	1.07E+06	g	8.78E+10	8.80E+16
2	Energy	3.33E+09	J		4.86E+14
2a	Electricity	1.42E+03	J	2.21E+05	3.14E+08
2b	Natural gas	3.33E+09	J	1.40E+05	4.86E+14
3	Services	6.88E+01	\$	2.02E+12	1.39E+14
4	Water	1.37E-01	m^3	3.26E+11	4.46E+10
5	Caustic soda (NaOH)	2.50E+04	g	4.14E+09	1.04E+14
	Total emergy				**8.88E+16**
	UEV for sulfur	Without capital invest		**8.88E+10**	sej/g S

Bold values are the total annual emergy inputs for 1 ton recovered sulfur and UEV for S.
Source: Koscienlnuk *et al.* (2010).

uses acid to decompose the PR. The most common acid used on wet process is sulfuric (although others such as nitric acid are also used) acid.

$$Ca_{10}F_2(PO_4)_6 + 10H_2SO_4 + 10nH_2O \rightarrow 10CaSO_4.nH_2O + 6H_3PO_4 + 2HF$$

By-product reuse and reclamation of mineland. In the production of wet-process phosphoric acid, by-products or tailing disposal materials are produced, which are slime and phosphogypsum. Slime is produced by the separation of clay and other ''fines'' from PR, which may be placed in settling ponds, disposed of in the sea, or placed in mined-out areas for

Supplementary Table S12. Emergy Evaluation of 1 Ton of Sulfuric Acid H_2SO_4 (100%)[a]

Note	Description	Data	Unit	UEV (sej/unit)	Emergy (sej/year)
Construction inputs					
	Capital	2.90E+00	\$	2.02E+12	5.86E+12
Operational inputs					
1	Elemental sulfur	3.30E+05	g	9.50E+10	2.93E+16
2	Energy				
2a	Electricity		J		
2b	Steam		g		
3	Services	9.50E+01	\$	2.02E+12	1.92E+14
4	Cooling and processing water	7.56E+01	m^3	4.96E+11	3.75E+13
	Total emergy				**2.95E+16**
	UEV for sulfuric acid	Without capital invest		**2.95E+10**	sej/g H_2SO_4
		With capital invest		**2.95E+10**	sej/g H_2SO_4

[a]Hydrogen sulfide is a by-product of processing natural gas and refining high-sulfur crude oils. No additional energy is needed for the process.
Bold values are the total annual emergy inputs for 1 ton H_2SO_4 and UEVs for H_2SO_4.
Source: UNIDO/IFDC (1998).

Supplementary Table S13. Emergy Evaluation of 1 Ton of Ammonia (NH_3)[a]

Note	Description	Data	Unit	UEV (sej/unit)	Emergy (sej/year)
Construction inputs					
	Capital	1.23E+01	$	2.02E+12	2.50E+13
Operational inputs					
1	Natural gas (fuel and feedstock)	3.79E+10	J	1.40E+05	5.53E+15
2	Services	5.41E+02	$	2.02E+12	1.10E+15
3	Cooling and processing water	2.12E+02	m^3	3.26E+11	6.91E+13
	Total emergy				**6.70E+15**
	UEV for ammonia	Without capital invest		**6.70E+09**	sej/g NH_3
		With capital invest		**6.72E+09**	sej/g NH_3

[a]Source: Shreve and Brink (1977); UNIDO/IFDC (1998).
Bold values are the total annual emergy inputs for 1 ton NH_3 and UEVs for NH_3.

reclamation. Phosphogypsum (PG) is produced in the reaction of sulfuric acid with PR, and can be used as construction material or converted to cement and sulfuric acid. A small percentage ($\sim 15\%$) of PG is reused in agricultural applications and plaster boards. Where nitric acid is used to produce P fertilizer, there is no gypsum by-product.

Mineland is reclaimed after ore is removed and is considered more productive than in the original state. Revegetation and reestablishment of ecosystem and wildlife habitat are of utmost importance in reclamation efforts.

Sulfur production and emergy values

Sulfur recovery refers to the conversion of hydrogen sulfide to elemental sulfur. Hydrogen sulfide is a by-product of processing natural gas and refining high-sulfur crude oils. Conversion of hydrogen sulfide (H_2S) to elemental sulfur by the Claus process has two steps (Supplementary Fig. S2):

(a) Thermal: the H_2S is partially oxidized in a furnace at high temperatures (1,000–1,400°C). Sulfur is formed and some H_2S remains:

$$2H_2S + 3O_2 \rightarrow 2SO_2 + 2H_2O + \text{heat}$$

(b) Catalytic: the remaining H_2S reacts with the SO2 at lower temperatures (about 200–350°C) over a catalyst to make more sulfur.

$$2H_2S\ +\ SO_2 \rightarrow 3S + 2H_2O + \text{heat}$$

Calculations assume a two-stage Claus unit with SuperClaus® and Dynawave®

Standard Claus Unit (one thermal and at least two catalytic steps).

SuperClaus unit recovers elemental sulfur from H_2S by suppressing SO_2 formation in the Claus stages, and selectively oxidizing H_2S in the presence of oxygen over a proprietary catalyst.

$$\text{SuperClaus Reaction}: H_2S + 1/2O_2 \rightarrow S + H_2O$$

Dynawave unit is a wet gas scrubber technology for emission regulation compliance.

Approximately 99% of the H_2S is captured and recovered as elemental sulfur by the SuperClaus and the remaining S is scrubbed and converted to Na_2SO_4 by the Dynawave. A sulfur recovery facility processes acid gas containing 77 mol% H_2S and 8 mol% NH_3.

Ammonia production and emergy values

The system for ammonia synthesis is based on the following reaction:

$$1.2N_2(g) + 3/2H_2(g) \rightarrow NH3(g)$$

This reaction is highly exothermic, and consequently, the design of the converter must be such to control the temperature at the point desired for the conversion deemed economical for the particular conditions chosen by the chemical engineers.

Ammonia synthesis gas is prepared by high-pressure catalytic reforming of hydrocarbon feed gas (e.g., natural gas) in the primary reformer with superheated steam, and in the secondary reformer with air to furnish nitrogen (N). The amount of air is adjusted eventually to supply an H_2/N_2 molar ratio of 3:1.

CO is converted by the catalytic iron oxide shift conversion with H_2O (from the reformer plus steam) to CO_2. CO_2 is removed solution wash (with reboiler regeneration yielding CO_2).

This is followed by methanation. The 3:1 hydrogen/nitrogen mixture, freed of its CO, is raised to the full compression of 150–200+ atm and missed with the recompressed, recirculated gases. The gases are then passed to the water secondary heat exchanger and ammonia refrigerator and separator for removal of residual ammonia.

In the ammonia converter, the gases are raised in a countercurrent heat exchanger to the reaction temperature and caused to react in the presence of a catalyst, after which they are cooled and most of the ammonia is liquefied.

Caustic soda manufacture. More than 95% of the capacity to produce chlorine and essentially 100% of the capacity to produce caustic soda (sodium hydroxide, NaOH) are based on the electrolysis of brine. In this process, a sodium chloride (NaCl, salt) solution (brine) is electrolytically decomposed to elemental chlorine (in the anode compartment), and sodium hydroxide solution and elemental hydrogen (in the cathode compartment). A chemical facility that coproduces caustic soda and chlorine is typically referred to as a chlor-alkali (C/A) facility. The overall reaction for the electrolytic production of caustic soda and chlorine is as follows:

$$2\ NaCl(aq) + 2\ H_2O \xrightarrow[\text{Electrical energy}]{} \frac{\text{Anode}}{Cl_2(gas)} + \frac{\text{Cathode}}{H_2(gas) + 2NaOH(aq)}$$

Supplementary Table S14. Emergy Evaluation of 1 Ton of Caustic Soda (50%) and 0.9 Ton of Chlorine[a]

Note	*Description*	*Data*	*Unit*	*UEV (sej/unit)*	*Emergy (sej/year)*
Construction inputs					
	Capital	2.93E+06	\$/year	2.02E+12	5.93E+18
Operational inputs					
1	Sodium chloride	1.50E+06	g	8.33E+08	1.25E+15
2	Energy	8.28E+09	J		1.75E+15
2a	Electricity	7.74E+09	J	2.21E+05	1.71E+15
2b	Fuels	5.40E+08	J	8.53E+04	4.61E+13
3	Services	5.59E+02	\$	2.02E+12	1.13E+15
4	Water	1.00E+01	m^3	3.26E+11	3.26E+12
	Total emergy				**4.14E+15**
	UEV for caustic soda	Without capital invest		**4.14E+09**	sej/g NaOH

[a]Source: Campbell and Ohrt (2009).
Bold values are the total annual emergy inputs for 1 ton chlorine and UEV for NaOH.

The electrolytic route to making caustic soda, by using sodium chloride as a raw material, is a coproduct process; for each 1.1 pound of sodium hydroxide produced, 1 pound of chlorine is also produced. This ratio is referred to as an electrochemical unit (ECU) (Campbell and Ohrt, 2009).

Major emergy carriers of fertilizer production

To better understand the allocation of total solar emergy for the DAP and struvite (Crystal Green) production process, a pie-chart representation is shown in Supplementary Fig. S3a and b, respectively. From Supplementary Fig. S3a, it is evident that elemental sulfur (84%) followed by PR (12%) in the DAP production contributes significantly to the total DAP emergy. Although the quantity of PR used in the process is greater than that of sulfur, the extraction of sulfur is an energy-intensive process and hence leads to higher transformity value. Magnesium chloride (93%) contributes majorly to the total emergy of struvite recovery process with higher quantity and transformity.

Supplementary References

Agrium, Inc. (2013). *DAP Diammonium Phosphate 18-46-0 (Bulk) US-GHS*. Available at: www.agrium.com/products/code/1527–25542 (accessed April 19, 2016).

Arden, S., Ma, X.C., and Brown, M. (2019). Holistic analysis of urban water systems in the Greater Cincinnati region: (2) resource use profiles by emergy accounting approach. *Water Res. X* 2:100012.

Bastianoni, S., Campbell, D.E., Ridolfi, R. and Pulselli, F.M. (2009). The transformity of petroleum fuels. *Ecol. Modell.* 220, 40.

Baur, R.J. (2009). Waste activated sludge stripping to remove internal phosphorus. US patent, 7604740B2.

Brown, M., and Ulgiati, S. (2002). Emergy evaluations and environmental loading of electricity production systems. *J. Clean. Prod.* 10, 321.

Brown, M.T., Campbell, D.E., De Vilbiss, C., and Ulgiati, S. (2016). The geobiosphere emergy baseline: A synthesis. *Ecol. Modell.* 339, 92.

Brown, M.T., Protano, G., and Ulgiati, S. (2011). Assessing geobiosphere work of generating global reserves of coal, crude oil, and natural gas. *Ecol. Modell.* 222, 879.

Campbell, D.E., Lu, H., and Walker, H.A. (2014). Relationships among the energy, emergy, and money flows of the United States from 1900 to 2011. *Front. Energy Res.* 2, 1.

Campbell, D.E., and Ohrt, A. (2009). Environmental Accounting Using Emergy: Evaluation of Minnesota. EPA/600/R-09/002. Washington, DC: U.S. Environmental Protection Agency.

Caruso, C., Catenacci, G., Marchettini, N., Principi, I., and Tiezzi, E. (2001). Emergy based analysis of Italian electricity production system. *J. Therm. Anal. Calorim.* 66, 265.

Cherubini, F., Raugei, M., and Ulgiati, S. (2008). LCA of magnesium production—Technological overview and worldwide estimation of environmental burdens. *Resour. Conserv. Recy.* 52, 1093.

Ciotola, R.J., Lansing, S., and Martin, J.F. (2011). Emergy analysis of biogas production and electricity generation from small-scale agricultural digesters. *Ecol. Eng.* 37, 1681.

Cohen, M.J., Sweeney, S., and Brown, M.T. (2007). Computing the unit emergy value of crustal elements. *The Fourth Biennial Emergy Conference*. Center for Environmental Policy, Department of Environmental Engineering Sciences, University of Florida, Gainsville, FL.

Hau, J.L., and Bakshi, R.B. (2005). Making emergy analysis more popular for environmentally concious design and manufacturing—Challenges and opportunities. *The Third Biennial Emergy Conference*. University of Florida, Gainesville, FL.

Ishii, S.K. (2015). Investigating the technical and social challenges of urine source separation from a life cycle perspective. Ph.D. dissertation. Gainesville, FL: University of Florida.

Koscienlnuk, D., Scheel, F., Meyer, S., Trapet, A., and Goar, G. (2010). *Low Cost and Reliable Sulfur Recovery*. Available at: https://vdocuments.mx/low-cost-reliable-sulfur-recovery.html (accessed June 25, 2018).

McClanahan, T.R. (1990). Hierarchial control of coral reef ecosystems. Ph.D. dissertation. Gainesville, FL: University of Florida.

McClellan, G.H. and Gremillion, L.R. (1980). The Role of Phosphorus in Agriculture. Madison, WI: American Society

of Agronomy, Crop Science Society of America, Soil Science Society of America.

Odum, H.T. (1996). *Environmental Accounting: Emergy and Environmental Decision Making*. New York, NY: John Wiley & Sons, Inc.

Odum, H.T. (May 2000). *Handbook of Emergy Evaluation—Folio #2 Emergy of Global Processes*. Gainsville, FL: Center for Environmental Policy, Environmental Engineering Sciences, University of Florida.

Ostara Nutrient Recovery Technologies, Inc. (2013). *Pearl and WASSTRIP Process*. Available at: http://ostara.com/wp-content/uploads/2017/03/Ostara_NRS_BROCHURE_170328.pdf (accessed March 8, 2018).

Paoli, C., Vassallo, P., and Fabiano, M. (2008). Solar power: An approach to transformity evaluation. *Ecol. Eng.* 34, 191.

Shreve, R.N., and Brink, J.A., Jr. (1977). *Chemical Process Industries*. New York: McGraw-Hill, 244.

UNIDO/IFDC. (1998). Fertilizer Manual. Muscle Shoals, AL: Kluwer Academic Publishers.

Vallero, A., Vallero, A., and Vieillard, P. (2012). The thermodynamic properties of the upper continental crust, Gibbs free energy and enthalphy. *Energy* 41, 121.
